# Interpretable machine learning for identifying ICU readmission risk in subgroups with probabilistic rules

**DOI:** 10.1093/jamia/ocaf171

**Published:** 2025-10-29

**Authors:** Lincen Yang, Siri L van der Meijden, Sesmu M Arbous, Matthijs van Leeuwen

**Affiliations:** Leiden Institute of Advanced Computer Science, Leiden University, 2333 CC, Leiden, The Netherlands; Department of Intensive Care Medicine, Leiden University Medical Center, 2333 ZA, Leiden, The Netherlands; Healthplus.ai B.V., 1013 NH, Amsterdam, The Netherlands; Department of Intensive Care Medicine, Leiden University Medical Center, 2333 ZA, Leiden, The Netherlands; Department of Clinical Epidemiology, Leiden University Medical Center, 2333 ZA, Leiden, The Netherlands; Leiden Institute of Advanced Computer Science, Leiden University, 2333 CC, Leiden, The Netherlands

**Keywords:** probabilistic rules, intensive care unit, readmission, subgroup analysis, interpretable machine learning

## Abstract

**Objective:**

Estimating readmission risk for intensive care unit (ICU) patients is critical for clinicians to optimize resource allocation and prevent premature discharges. Machine learning models currently applied to this task either lack interpretability or cannot identify patient subgroups with distinctive readmission risks and characteristics. We addressed this gap by introducing a cutting-edge rule-based model, namely truly unordered rule sets (TURS), to reveal heterogeneous readmission risks and subgroup-level patient characteristics.

**Materials and methods:**

We trained TURS on all ICU admissions from January 2011 to January 2020 at Leiden University Medical Center. For each subgroup, patient characteristics and the influence of feature variables on readmission risk were analyzed.

**Results:**

TURS identified subgroups with heterogeneous feature distributions and feature importance, providing actionable insights for ICU discharge planning. Its predictive performance (area under the receiver operating characteristic curve [ROC-AUC] 70.5%) and model complexity (5 rules, average length 2) surpassed other rule-based models.

**Discussion:**

Subgroup analysis highlighted the heterogeneity of patients. First, we compared the conditional probability distribution of each feature variable, conditioned on the fact that a patient was in a certain subgroup, with its unconditional distribution. We identified features deviating from the unconditional distribution, illustrating unique subgroup-specific implications. Furthermore, we demonstrated that features with the highest impact on the readmission risk were distinctive for each subgroup.

**Conclusion:**

The TURS model provided a concise summary of patient subgroups, aiding ICU discharge decisions and advancing knowledge discovery in the ICU.

## Introduction

In intensive care units (ICU), deciding whether to discharge a patient to the hospital’s wards is a daily challenge. The scarcity and costliness of ICU resources require efficient management by discharging patients as soon as safely possible. However, premature discharge is irresponsible for patients, as this may lead to readmission to the ICU, which is correlated to increased mortality, morbidity, costs, and prolonged hospital stays.^1^^,^^2^ Approximately 5.9% of patients^3^ are readmitted, which motivates leveraging machine learning models to predict ICU readmission risk for patients before they are discharged.^4–6^ Such a data-driven approach may reduce human errors in decision-making by offering precise predictions based on similar patients’ histories, complementing physicians’ assessments and enhancing their decision-making process.^7^

While black-box models like neural networks and tree boosting achieve high predictive performance,^5^^,^^6^^,^^8^ they lack transparency and hardly reveal human-comprehensible patterns,^9^ making it difficult for clinicians to understand and trust their predictions. Meanwhile, existing interpretable models have their own drawbacks. For instance, linear models (eg, logistic regression), though widely used in the healthcare domain,^10–14^ suffer from the over-simplified linearity assumption. Furthermore, to explicitly characterize heterogeneity of patients, clustering-based methods (also referred to as phenotyping)^4^^,^^15^^,^^16^ are widely used. However, these unsupervised learning methods ignore the association between the target variable (eg, readmission or not) and the patients’ features.

By contrast, classification rules,^17–22^ a supervised machine learning approach, can partition ICU patients into subgroups with distinctive readmission risks such that each subgroup can be identified by a human-understandable rule. Classification rules offer concise summaries of different patient types, allowing ICU physicians to understand the conditions associated with varying readmission risks and make more informed decisions.

Nevertheless, despite its great potential in discovering human interpretable patterns about patients, classification rules are under-explored in healthcare applications, which we conjecture is due to the following technical drawbacks. To begin with, although rule-based methods have been an active research field for decades, recently proposed methods focus on non-probabilistic rules,^23–26^ which do not apply to our task as the readmitted patients are only a small proportion and hence the class labels are highly imbalanced. Furthermore, classification rules often come with an order (rank) of the rules, which can seriously hamper the interpretability of the model. In other words, while a single classification rule is directly readable by humans and hence highly interpretable, a rule set model formalized by putting multiple rules together (as one single rule is hardly enough to characterize all cases) can be very challenging to interpret, on which we will elaborate with examples in “Motivation for TURS” section.

This paper aims to address the aforementioned limitations of interpretable machine learning models by leveraging a cutting-edge probabilistic rule set model, the truly unordered rule set (TURS).^27^ First, we applied TURS to identify a highly interpretable rule set distinguishing subgroups of patients with different ICU readmission risks. Second, we compared the predictive performance of the TURS model to commonly used rule set models and other state-of-the-art machine learning models. Third, we conducted in-depth subgroup analysis and demonstrated that different subgroups of patients were highly heterogeneous in their characterizations, justifying the necessity of conducting subgroup-specific analysis. Finally, for each subgroup of ICU patients, we modeled the associations between their readmission risk and attributes. As a result, we identified subgroup-specific important factors that greatly contributed to a patient’s risk of being readmitted or not to the ICU.

## Methods

In the following, we discuss the dataset, model development, and the analysis methods.

### Data and participants

#### Data source

The dataset was collected at the ICU of Leiden University Medical Center (LUMC), a 900-bed tertiary academic hospital in the Netherlands. All admissions to the ICU between January 2011 and January 2020 were included. We extracted and pseudo-anonymized observational patient data from the Patient Data Management System (PDMS, MetaVision version 5 and 6, IMDsoft, Tel Aviv, Israel) and the Electronic Health Record (EHR, HiX, Chipsoft, Amsterdam, The Netherlands). The original dataset is multi-modal and contains information in different forms, including time series measurements (eg, cardiology monitor records), lab results over time (eg, blood tests), medication use records, as well as static information for each patient (eg, gender).

#### Participants

We evaluated all adult (≥18 years) ICU admissions to train and validate models. ICU admissions shorter than 12 hours and admissions with a direct transfer from the ICU to another hospital were excluded from the analysis. We included admissions with successful discharge to the general ward, excluding patients who deceased in the ICU. Data regarding do-not-resuscitate and do-not-intubate orders were unavailable. Consequently, these admissions could not be excluded from analysis, which has potentially resulted in bias as these patients will not be readmitted to the ICU. We did not take into account admissions starting in 2020, due to changing circumstances influenced by COVID-19.

#### Outcome

The predicted outcome was defined as readmission to the ICU or Medium Care Unit within 7 days after discharge from the ICU to a general hospital ward.

#### Included predictors and feature engineering

The dataset was pre-processed into a tabular dataset. Variables could be divided into static variables (patient characteristics) and time-series variables (laboratory results, vital signs, and medication data). Time-series variables were measured multiple times during an admission, with different sample frequencies. Feature engineering was performed to capture descriptive aspects of time series variables. For this purpose, 3 time-windows were used: the first 24 hours of the admission, the last 24 hours of the admission before discharge, and the full admission period. For each time window, several aggregates were calculated: the mean, standard deviation, minimum, maximum, and count (number) of measurements. Furthermore, a feature was created for when a variable was missing to capture missingness-not-at-random.

We distinguished patient variables into 7 categories for further analysis: (1) respiratory (eg, respiratory rate, ventilator machine settings); (2) circulatory (eg, blood pressure); (3) hematological (eg, hemoglobin); (4) metabolic (eg, lactate); (5) kidney (eg, urea); (6) liver (eg, bilirubin); and (7) patient characteristics (eg, gender, the location a patient is admitted from such as own home, elderly home, or other hospitals).

#### Missing data and pre-processing

We excluded variables with more than 50% missing data that could not be explained by domain knowledge of parameters missing not at random. Imputation of remaining missing values was performed using mean imputation for numerical variables and mode imputation for categorical features. Mean/mode imputation is a simple and efficient method, and can be applied to the data despite its high dimensionality and large size. In contrast, methods like k-NN imputation^28^ may suffer from instability in high dimensions,^29^ where distance metrics become less meaningful—a phenomenon known as the curse of dimensionality.^30^ This issue is further compounded when dealing with mixed variable types, where defining a unified and meaningful distance metric is nontrivial. Furthermore, MICE^31^ is computationally expensive due to its iterative nature and may be unstable, as uncertainty from sequential predictions can accumulate over iterations.^32^^,^^33^

Categorical features (eg, the location a patient is admitted from such as own home, elderly home, or other hospitals) were transformed into numerical data using one-hot encoding. Continuous numerical features (eg, age, respiratory rate) were standard-scaled with zero mean and unit variance. Feature engineering of the included variables resulted in a total of 550 features per patient.

### Model development

We first review probabilistic rule sets and meanwhile describe the notation, and then we introduce the TURS model.

#### Probabilistic rules

A probabilistic rule takes the form of “*If X meets a certain condition, then* $Y∼\hat{P}(Y)$.” Formally, a condition is defined as logical conjunctions of *literals*, in which each literal is a tuple that consists of (1) a feature variable; (2) an operator (ie, “≥,” “ < ,” or “=”); and (3) a value. For instance, a literal for ICU patients may be {age≥30}, and a rule condition consists of one or multiple literals, connected by the logical “And.” Furthermore, *X* denotes a vector of feature values, and $\hat{P}(Y)$ denotes the probability estimation for the outcome (readmission risk) *Y*, ie, the empirical proportions of readmitted patients.

A rule identifies a subgroup of patients, for which we define the *cover* of a rule as the patients that satisfy its condition, and the *coverage* as the number of these patients. Learning a probabilistic rule from data concerns the trade-off between accuracy and coverage: a desired rule should be specific and small enough to accurately estimate P(Y), yet the coverage should be large enough to ensure that $\hat{P}(Y)$ can be estimated reliably from data.

#### Rule sets

A probabilistic rule set model *M* consists of multiple probabilistic rules, which we denote as $M=\{S_{1},\ldots ,S_{K}\}$. Each $S_{k}$ represents a single probabilistic rule for k∈{1,…,K}. A rule set identifies different subgroups of patients, which can be distinctive from each other in terms of readmission risks and underlying risk factors as characterized by their features.

Furthermore, given a fixed model *M* and an ICU patient, with their feature vector denoted as $\vec{x}$, estimating their readmission risk $P(Y=y|X=\vec{x})$ involves 2 steps. First, we identify the rules that cover the patient’s features $\vec{x}$. Second, if only one rule, denoted as *S*, covers $\vec{x}$, then $P(Y=y|X=\vec{x})$ is predicted by the empirical proportion of readmissions estimated from all patients covered by *S*.

However, if multiple rules cover the patient’s feature vector $\vec{x}$, rules are said to be overlapped and each rule may predict a different readmission risk $\hat{P}(Y)$. Specifically, the way that traditional rule-based methods tackle this challenge notoriously complicate the comprehension and interpretation of each probabilistic rule, as we elaborate next.

#### Motivation for TURS

Traditional approaches tackle this challenge mostly by imposing an order (rank) among the rules and following the probability estimation given by the highest-ranked rule,^23^^,^^24^ in which the ranks are defined by some quality measure for individual rules (eg, the accuracy or F-1 score).

We next explain how ranks among rules may hamper the interpretability of the whole model.^27^^,^^34^ For illustration, consider as an example the rules to classify animals, in which the rule is ranked and connected with “if—else if—…” statements:

**Table:** 

**If** Having-feather = True	**Then** Bird;
**Else if** Number of legs = 2	**Then** Chimpanzee;
…	

Notably, the second rule must be interpreted under the condition that the first rule is not satisfied, otherwise it would not be a meaningful explanation for the prediction “chimpanzee.”

As illustrated in the example, rules must be interpreted by taking into account all higher-ranked rules in general. Yet, when the number of rules becomes large, it is infeasible for a human domain expert to go over all higher-ranked rules.

Although some existing methods claim to learn unordered rule sets from data, they still (implicitly) impose ranks among rules to handle the conflicts when multiple rules apply to one instance. For instance, consider the rules modelling flight delays shown below:

**Table:** 

**If** Weather = storm	**Then** Flight is delayed;
**If** Flight duration >10 hours	**Then** Flight is on time.

Potentially, when both rules apply to one single instance (a flight), we need to decide which rule to use to make the final prediction. Most existing methods^23^^,^^24^^,^^19^^,^^35^ define a “quality” measure for rules (eg, how accurate a rule is) and always follow the one with higher “quality,” which (implicitly) puts a rank among all rules as well.

Consequently, although single probabilistic rules are directly readable by clinicians, a rule set as a whole is difficult to comprehend by clinicians, which hinders its wide application in healthcare domains. To overcome this shortcoming, we introduce the cutting-edge “Truly Unordered Rule Set (TURS)”^27^^,^^22^ to the task of ICU readmission risk prediction.

#### Truly unordered rule sets

Given a rule set $M=\{S_{1},\ldots ,S_{K}\}$ that contains *K* probabilistic rules, the conditional probability $P(Y=y|X=\vec{x})$ of readmission, denoted as Y∈{0,1}, conditioned on the patients feature variables $\vec{x}$ is defined as follows. First, we identify the subset of rules ${\left\{S_{j}\right\}}_{J}\subset M$ that covers $\vec{x}$, in which *J* is a subset of the index set {1,…,K}. Next, the conditional probability is estimated as


(1) $$P(Y=y|X=\vec{x})=\frac{\left|\{(X,Y)|Y=y,X\in \cup_{j\in J}S_{j}\}\right|}{\left|\{(X,Y)|X\in \cup_{j\in J}S_{j}\}\right|},$$

in which |.| denotes the cardinality of a set. Hence, the denominator represents the number of patients covered by the union of ${\left\{S_{j}\right\}}_{J}$. Within the union of these subgroups identified by ${\left\{S_{j}\right\}}_{J}$, the numerator represents the number of patients with the target variable Y=y.

Taking the union for modelling the overlap is a deliberate design choice that leads to compelling consequences. Specifically, such a formalization favors “overlapping” subgroups to have “similar” readmission risks.^27^ Furthermore, it has been empirically shown through a large-scale benchmark that when a data point is covered by multiple rules, the class probability estimates are so similar in practice that one could even randomly pick and use one of the overlapping rules (instead of all of them).^22^

Thus, this model formalization avoids the necessity for ranking rules or averaging the predictions given by different rules. As a result, the readmission risk for the patient subgroup identified by a probabilistic rule is valid for all individual patients within this subgroup, making the individual probabilistic rules self-standing and meanwhile making the rule set truly unordered.

#### Predictive performance evaluation

We divided the total dataset randomly into a training and test dataset, which contains 9737 and 2435 patients respectively (approximately 80%/20% splitting). Furthermore, the predictive performance was evaluated on the test dataset according to the area under the receiver operating characteristic curve (ROC-AUC) and the area under the precision-recall curve (PR-AUC). 95% confidence intervals were generated using 1000 sample bootstrapping with replacement.

The TURS model was compared to several widely used rule-based methods and commonly used black-box random forest and XGBoost machine learning models. Rule-based models included CN2,^34^ CART,^36^ RIPPER,^35^ and C4.5.^19^

### Subgroup analysis

Besides the predictive performance, we evaluate the potential of TURS by investigating the heterogeneity between different patient subgroups. Specifically, we adopt logistic regression separately for each subgroup to identify important features that have critical influences on readmission, which aims to discovery insights such as “The readmission risk is highly sensitive to circulatory variables for one subgroup but not another.” See Appendix SI for more details on how Logistic regression with L1 regularization was applied.

Furthermore, we presented data visualizations for exploring heterogeneous patient characteristics among different subgroups. Through visualizations, we aimed to identify features with “deviating” probability distributions, by comparing the distribution of each feature variable within the subgroup to the whole group of all patients. For instance, if we observe that one patient subgroup has substantially higher lactate counts than the whole patient cohort (despite that the lactate counts is not part of the rule condition), such a property provides additional insights for understanding the patient characteristics of this particular subgroup. See Appendix SII for more details on how the characterizing deviating features were determined.

## Results

In this section, the patient characteristics of the dataset, the results of the TURS model, and the subgroup analysis are presented.

### Participants and inclusion of ICU admissions

We identified 15 749 ICU admissions in the dataset, of which 12 172 could be included for model development and testing. The overall readmission rate was 6.71% (*n* = 852). Main reasons for exclusion were admissions shorter than 12 hours (*n* = 1223), patients that did not survive their ICU admission (*n* = 1600), and direct transfer from the ICU to another hospital (*n* = 651). (A flowchart with the reasons for other reasons for exclusion is provided in Appendix SIII.) Patient characteristics are shown in Table 1. Readmitted patients had a significantly higher mortality rate compared to not-readmitted patients (19.83% vs 4.41%, P<.001). Furthermore, readmitted patients were significantly more often emergency admissions, general surgical patients, internal medicine patients, and significantly less often thoracic surgical patients.

**Table 1.** Patient characteristics: comparison of readmission and no readmission groups.

	Readmission (%)	No readmission (%)	*P*
Patients	6.71	93.29	—
Women	36.98	34.53	.164
30-day mortality	19.83	4.41	.000
Vasoactive drugs use	68.73	67.82	.616
Priority code (emergency admission)	49.51	36.8	.000
General surgical patients	21.17	11.45	.000
Thoracic surgical patients	31.14	56.27	.000
Internal medicine patients	8.64	6.53	.024
Neurosurgical patients	9.85	8.06	.081

### The learned TURS model

We obtained a very concise rule set that summarizes all patients into 5 subgroups, as shown in Table 2. Based on our domain knowledge of ICU patients, we also have included the clinical implication/explanation of each rule as “clinical annotations.”

**Table 2.** The TURS model learned from the ICU dataset.

Rule no.	Rule conditions	Prob. readmission	No. of patients
$S_{1}$	Urea (max/all) ≥ 12.1 *AND*	.223	494
	Respiratory Rate (median/last24h) ≥ 23.5		
	(*Clinical annotation: Elevated urea and respiratory*		
	*rate may indicate renal dysfunction in the context*		
	*of a compensatory respiratory response and/or*		
	*respiratory failure. It could also indicate a*		
	*combination of renal dysfunction and respiratory failure.*)		
$S_{2}$	APTT (max/all) ≥ 43.1 *AND*	.199	548
	Urea (mean/all) ≥ 16.338		
	(*Clinical annotation: Prolonged APTT and elevated urea*		
	*may suggest a combination of coagulopathy and renal*		
	*impairment, possibly in the context of multi-organ failure.*)		
$S_{3}$	Leukocytes (mean/last24h) ≥ 20.81	.162	464
	(*Clinical annotation: Elevated leukocytosis may*		
	*indicate* (*severe*) *systemic inflammation or*		
	*infection.*)		
$S_{4}$	Potassium (count/first24h) ≥ 6.0 *AND*	.131	1979
	Patient is NOT admitted after cardiothoracic surgery		
	(*Clinical annotation: Frequent bloodgas measurements*		
	*may reflect clinical instability* (*both respiratory or*		
	*circulatory*) *or concern for electrolyte disturbances in*		
	*non-cardiothoracic patients.*)		
$S_{5}$	Platelets (count/first24h) ≥ 2.0 *AND*	.019	3922
	Urea (last/last24h) < 9.2 *AND*		
	Patient is admitted after cardiothoracic surgery		
	(*Clinical annotation: Frequent platelet measurements*		
	*with normal to low urea post-cardiothoracic surgery*		
	*may reflect routine monitoring in stable patients.*)		
$S_{d}$	None of the above	.059	3220
	(*Clinical annotation: All patients not falling into one*		
	*of the above groups.*)		

The rule conditions can be interpreted as “variable (aggregation/timewindow),” in which 3 time-windows were used, including the first 24 hours of the admission (*first24h*), the last 24 hours of the admission before discharge (*last24h*), and the full admission period (*all*). For each time window, different aggregation methods were applied, which includes maximum (*max*), mean, the number of measurements (*count*), and the last measurement (*last*). Last, APTT stands for activated partial thromboplastin time.

The highest probability of readmission was observed in subgroup $S_{1}$. In this group, a high maximum measurement of urea, indicating kidney malfunction, and a high respiratory rate during the last 24h before discharge, indicating respiratory distress, all together lead to a probability of readmission of 22%. Subgroup 2 had a probability of readmission of 20%, and was characterized by a lengthened APTT (activated partial thromboplastin time) and elevated urea. Patients in Subgroup 3, with elevated leukocytes in the last 24h of discharge, a marker for infection, had a probability of readmission of 16%. Subgroup 4, with a probability of 13%, had a higher number of potassium measurements done in the first 24h of ICU admission, and were not admitted to the ICU after cardiothoracic surgery. A low readmission probability of 2% in Subgroup 5 was characterized by a low number of thrombocytes measurements, a last measured urea value < 9.2 before discharge, and being admitted because of cardiothoracic surgery. Patients not satisfying any of the 5 rules had a 6% risk of readmission, which is close to baseline.

### Predictive performance comparison

We first evaluated the quality of the TURS model by its predictive performance on our dataset, reporting the ROC-AUC and PR-AUC scores on the test set in Table 3.

**Table 3.** Predictive performance and model complexities of rule-based methods when applied to the ICU dataset.

Method	ROC-AUC (95% CI)	PR-AUC (95% CI)	No. rules	Avg. rule length
TURS (ours)	0.705 (0.666, 0.746)	0.164 (0.124 0.206)	5	2.0
CN2	0.647 (0.603 0.688)	0.122 (0.093 0.166)	846	2.5
CART	0.686 (0.644 0.724)	0.138 (0.107, 0.178)	27	4.2
Ripper	0.504 (0.486, 0.503)	0.071 (0.056, 0.077)	2	6.0
C4.5	0.561 (0.509, 0.614)	0.094 (0.056, 0.095)	250	15.9
RF	0.727 (0.685 0.766)	0.175 (0.133 0.224)	—	—
XGBoost	0.752 (0.716, 0.785)	0.181 (0.140,0.234)	—	—

We also include the widely used black-box models random forest (RF) and XGBoost as (upper) baselines.

Abbreviations: CI, confidence interval; PR-AUC, area under the precision recall curve; ROC-AUC, area under the receiver operating characteristic curve .

The ROC-AUC measures how well our model distinguishes between readmitted and non-readmitted patients. If a rule is too generic, covering a large number of patients, the readmission risk estimate becomes imprecise, reducing the model’s discriminative power. Conversely, if a rule is too specific, covering too few patients, the readmission risk estimate becomes unreliable and cannot generalize well to new data, which also hurts the overall model performance.

The learned TURS model had the highest ROC-AUC and PR-AUC, in comparison to several widely used rule-based methods. TURS did not exceed the predictive performance of black-box random forest (RF) and XGBoost machine learning models.

The TURS model had the lowest model complexity of the rule-based models, measured by the number of rules and the rule lengths (ie, the average number of literals in each rule), with RIPPER as the only exception. However, RIPPER barely learned any informative structure from the data as shown by a ROC-AUC score close to 0.5.

### Subgroup analysis

#### Important risk factors for patient subgroups

While a rule set model can provide a concise summary for subgroups of patients, analyzing the readmission risks for individual patients requires comprehensive clinical interpretation. In Figure 2, we visualized the coefficients of the logistic regression model that was learned for each subgroup of patients. The number of features with non-zero coefficients for each subgroup (from $S_{1}$ to $S_{5}$ shown in Table 2) were highly heterogeneous; ie, 30, 22, 2, 56, and 14, respectively.

Per rule, it can be seen that different feature groups are important. For example, in subgroup $S_{1}$, the kidney function, specialty, and coagulation function are important variables, while in subgroup $S_{4}$, a patient’s liver function and length of stay (LOS) are important. For subgroups $S_{1}$, $S_{3}$, and $S_{5}$, some categories, such as infection, are not important risk factors for the readmission probability.

#### Deviating feature analysis

Table 4 characterizes, for each subgroup, the distributions of feature variables that deviate from that of the population. While each patient subgroup can be separated from the rest with the corresponding rule condition, other (correlated) variables provide further characteristics of each subgroup.

**Table 4.** Deviating features for each patients subgroup.

Rule	Category	Feature	Subgroup	Default
IF Urea (max/all) ≥12.1 AND Respiratory rate (median/last24h) ≥23.5 THEN Pr(Readmission) = 0.223	Patient characteristic	LOS	9.6 [4.4, 17.8]	1.1 [0.9, 2.9]
	Respiratory	Respiratory rate (median/last24h)	26.5 [25.0, 28.5]	17.0 [15.0, 20.0]
	Circulatory	Noradrenaline (total time)	2.3 [1.4, 5.9]	1.2 [0.3, 1.4]
	Coagulation	APTT (max/all)	47.1 [39.7, 69.9]	35.9 [32.0, 42.6]
	Liver	Bilirubin total (max/all)	21.0 [11.0, 30.0]	12.0 [8.0, 22.3]
	Kidney	Urea (max/all)	22.2 [16.2, 33.8]	7.5 [5.6, 11.7]
	Metabolic	BE arterial (max/all)	8.0 [4.4, 11.6]	0.0 [-2.0, 3.9]
	Infection	CRP (max/all)	251.9 [148.3, 338.5]	94.0 [50.6, 178.0]
IF APTT (max/all) ≥43.1 AND Urea (mean/all) ≥16.338 THEN Pr(Readmission) = 0.199	Patient characteristic	LOS	7.1 [3.0, 14.7]	1.1 [0.9, 2.9]
	Respiratory	Respiratory rate contr. (count/all)	18.0 [9.6, 20.8]	9.6 [5.0, 13.0]
	Circulatory	Noradrenaline (sum)	28.2 [13.7, 108.9]	16.1 [1.6, 26.2]
	Coagulation	APTT (max/all)	58.1 [48.6, 85.3]	35.9 [32.0, 42.6]
	Liver	Bilirubin Total (max/all)	22.3 [13.0, 40.0]	12.0 [8.0, 22.3]
	Kidney	Urea (max/all)	33.0 [25.0, 43.7]	7.5 [5.6, 11.7]
	Metabolic	Lactate (max/all)	3.4 [2.3, 5.6]	2.1 [1.5, 3.1]
	Infection	CRP (max/all)	242.0 [140.1, 334.9]	94.0 [50.6, 178.0]
IF Leukocytes (mean/last24h) ≥20.81 THEN Pr(Readmission) = 0.162	Patient characteristic	Specialty: ICCHI	0.211	0.121
	Respiratory	Respiratory rate (median/first24h)	17.5 [15.0, 21.0]	16.0 [14.0, 19.0]
	Circulatory	Noradrenaline (binary discharge)	0.034	0.014
	Coagulation	Thrombocytes (mean/last24h)	235.5 [174.1, 334.6]	183.0 [137.3, 235.5]
	Liver	ALAT (max/all)	48.0 [22.0, 115.2]	32.0 [19.0, 115.2]
	Kidney	Urea (min/last24h)	8.4 [5.9, 13.5]	6.7 [4.9, 9.6]
	Metabolic	Lactate (mean/last24h)	1.5 [1.2, 1.9]	1.3 [1.0, 1.6]
	Infection	Leukocytes (mean/last24h)	23.5 [21.8, 26.2]	12.2 [9.5, 14.9]
IF Potassium (count/first24h) ≥6.0 AND Patient is NOT admitted after cardiothoracic surgery THEN Pr(Readmission) = 0.131	Patient characteristic	Priority code 1	0.743	0.379
	Respiratory	Respiratory rate contr. (count/all)	14.8 [9.6, 19.8]	9.6 [5.0, 13.0]
	Circulatory	ABP systole (count/first24h)	23.0 [21.0, 24.0]	21.0 [18.0, 23.0]
	Coagulation	APTT (mean/first24h)	36.8 [32.0, 44.3]	32.5 [29.7, 36.5]
	Liver	Bilirubin Total (max/all)	16.0 [9.0, 25.0]	12.0 [8.0, 22.3]
	Kidney	Urea (max/all)	12.7 [7.9, 21.8]	7.5 [5.6, 11.7]
	Metabolic	BE arterial (max/all)	5.0 [0.7, 9.0]	0.0 [-2.0, 3.9]
	Infection	CRP (max/all)	165.1 [82.5, 291.1]	94.0 [50.6, 178.0]
IF Thrombocytes (count/first24h) ≥2.0 AND Urea (last/last24h) < 9.2 AND Patient is admitted after cardiothoracic surgery THEN Pr(Readmission) = 0.019	Patient characteristic	Specialty: ICCTC	1.000	0.547
	Respiratory	Respiratory rate (mean/all) missing	0.019	0.015
	Circulatory	Milrinone (time fraction)	0.5 [0.5, 0.5]	0.5 [0.5, 0.5]
	Coagulation	Hb (max/last24h)	6.9 [6.2, 7.6]	6.7 [5.8, 7.5]
	Liver	ASAT (min/all)	42.0 [31.0, 59.0]	47.0 [30.0, 73.6]
	Kidney	Urea (mean/all) missing	0.0	0.006
	Metabolic	Potassium (mean/all)	4.4 [4.2, 4.6]	4.2 [3.9, 4.5]
	Infection	Leukocytes (max/last24h)	13.6 [11.0, 16.6]	13.1 [10.0, 16.2]

For binary deviating features, the probabilities of taking the value “true” are reported; for numeric deviating features, the medians [IQR] are reported. As in Table 2, the Variable Names are represented in the format of “Variable (aggregation/timewindow).” Abbreviations (LOS, APTT, CRP, ALAT, Specialty: ICCHI, and Specialty: ICCTC) are explained in the Appendix SIV.

This can for example be seen in subgroup $S_{1}$ (Urea (max/all) ≥ 12.1 *AND* Respiratory Rate (median/last24h) ≥ 23.5), where patients had a longer median ICU length of stay (LOS), higher median respiratory rate, and a higher maximum CRP measurement throughout the admission. Furthermore, we also demonstrated that each subgroup of patients had different feature variables that deviated most from the population.

## Discussion

We introduced the TURS, a cutting-edge rule-based machine learning model, to the task of ICU readmission risk prediction.

We learned an interpretable model that predicts the readmission risk of ICU patients based on a wide range of patient features (from 7 categories, ie, patient characteristics, respiratory, circulatory, hematological, metabolic, kidney, and liver). The learned model outputs probabilistic predictions to overcome the highly imbalanced class labels (“only” 7% of patients were re-admitted). The learned rule sets also outperforms other baseline methods we compared to, both in terms of both predictive performance measured by ROC-AUC and model complexity (the average rule lengths and the number of rules).

Furthermore, the rule sets are highly interpretable because physicians can directly read the rules to understand the decision logic, and, more importantly, unlike previous rule-based methods, the learned model does not contain any orders among rules. As a result, rules can be comprehended independently, without having to exclude the scenarios described by higher-ranked rules.

The learned rule sets automatically and explicitly summarize *patient subgroups* that reveal heterogeneity among patients. In clinical and epidemiological studies, subgroups are commonly formalized manually by domain experts (eg, based on a certain age group, or a subpopulation with a certain risk factor such as smokers), often because the characteristics of these subgroups are known to deviate from those of the population. We identified 5 patient subgroups in a purely data-driven and automated manner.

We analyzed the heterogeneity of patients revealed by identified patient subgroups from the following perspectives. First, we examined the distribution of each feature conditioned on patient subgroup membership, and specifically, how much this conditional distribution deviates from the feature’s unconditional distribution. Deviating features characterize a patient subgroup by revealing the *implications* of belonging to a certain subgroup; for instance, as shown in Table 4, the average length of stay (LOS) for the first patient subgroup is 9.6 days, which is much higher than in the whole patient population (1.1 days). We demonstrated that, although each subgroup can be identified by a concise rule, the implications of belonging to each subgroup are considerable and distinctive.

**Figure 1.**
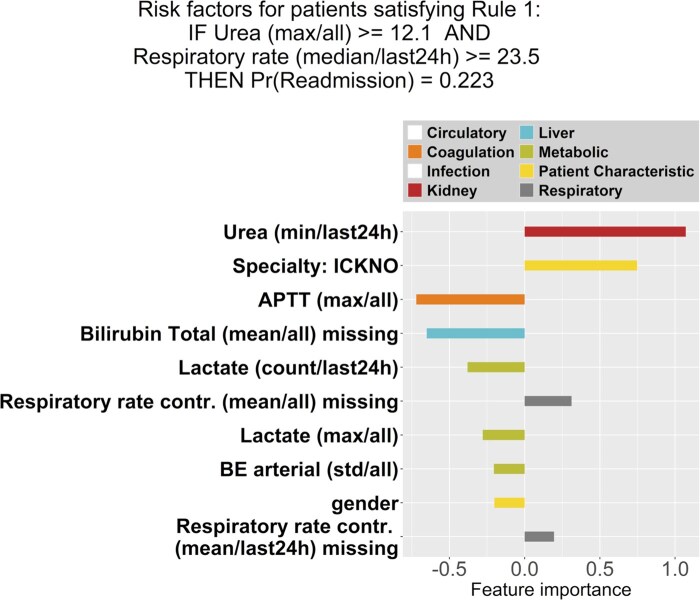
A logistic regression model is fit to the data of patients for Subgroup 1. The top-10 non-zero coefficients are shown, demonstrating the most influential patient’s features with regard to the estimated ICU readmission risk, as well as their corresponding feature importance. Features are categorized and presented by different colors as shown in the legend. If none of the top-10 important features belong to certain categories (Circulatory and Infection), the categories are shown as “white” in the legend. Variable names contain the information of feature engineering (if any), which are represented in the format of “Variable (Aggregation/Time Window).” Aggregations methods applied here include the first/last measurement within the time window (*last 24h*), the mean, standard deviation (*std*), minimum (*min*), maximum (*max*), and number of measurements (*count*). Abbreviations of the variable names are explained in Appendix SIV.

**Figure 2.**
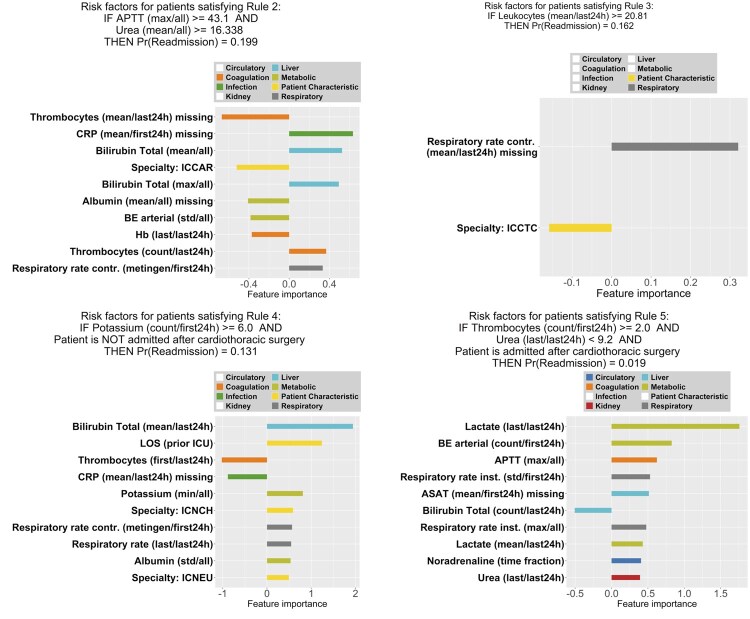
A logistic regression model is fit to the data of patients for Subgroup 2 to 5. The top-10 non-zero coefficients are shown (Subgroup 3 has only 2 non-zero coefficients), demonstrating the most influential patient’s features with regard to the estimated ICU readmission risk. Different colors are used to represent the categories of the features, and we observe heterogeneity of influential features for different subgroups. Variable names contain the information of feature engineering (if any), which are represented in the format of “Variable (Aggregation/Time Window).” Aggregations methods applied here include the first/last measurement within the time window (*first or last 24h*), the mean, standard deviation (*std*), minimum (*min*), maximum (*max*), and number of measurements (*count*). Abbreviations of the variable names are explained in Appendix SIV.

Second, we investigated heterogeneity in feature importance, ie, the features that are highly impactful to the probability of readmission, and we concluded that the feature variables/categories that are important to the patient subgroups are highly distinctive as well. For instance, in Figure 1, we observe for patients in Subgroup 1 that the most important feature belongs to the “kidney function” category, which is different from the remaining four subgroups as shown in Figure 2. In addition, feature variables related to infection have high influence on the readmission risk of patient subgroups 2 and 4, yet seem irrelevant for the other patient subgroups.

Our approach is different from clustering-based (phenotyping) approaches, which are commonly used for heterogeneity analysis in clinical studies.^4–6^ Clustering-based methods assess similarity between patients based only on their feature variable values, ignoring the associations between the feature and target variables. This can be problematic, since patient feature variables may be irrelevant to the ICU readmission risk yet influence whether two patients are similar enough to be clustered together. By contrast, our rule-based approach explicitly models the association between the feature variables and the target variable.

Traditional rule-based models suffer from drawbacks such as being incapable of making probabilistic predictions and interpretability issues caused by overlaps and orders among rules. By employing TURS and obtaining rules that can be interpreted independently and regardless of other rules in the model, we are the first to introduce rule-based methods both for interpretable predictions of ICU readmission risks and for identifying distinctive patient subgroups. Notably, rule-based approaches allow subgroup probabilities to be easily verified with new data, making them more trustworthy. In contrast, black-box models predict individual probabilities but are hard to confirm without identical data points.

We next discuss limitations and future research directions. First, due to missing values, not all relevant variables for ICU patients were included. For example, relevant non-clinical information such as socioeconomic status, access to follow-up healthcare and patient and family support systems was not available while being known as determinants to a patient’s readmission risk.^37^ Besides, the data collected in the ICU is multi-modal, and the pre-processing of time series data (including repeated measures over time) may cause information loss. With the continued development of large (language) models, pre-processing, and feature engineering for (multi-modal) clinical data may possibly be conducted automatically in the future,^38^ potentially leading to lower information loss.

Second, despite outperforming existing rule-based models, the predictive accuracy obtained with TURS is (slightly) below that of black-box models. This indicates that complex patterns may exist in the data that are difficult to capture by rule-based models. While black-box models are becoming more frequently implemented in a clinical setting,^39^ there is a debate on whether we should instead aim for using simpler, more interpretable models such as TURS for high-stake decision-making.^40^

Last, no user study has been conducted to assess the acceptance of rules learned from data. Such a user study is a necessary step towards deploying TURS as a decision support system in the ICU. Future work should therefore focus on user interface design and conduct clinician usability studies to evaluate whether interpretable models enhance trust and adoption in clinical practice.^41^

## Conclusion

In conclusion, the learned TURS model provides an interpretable, concise, and precise summary of patient subgroups, which may be used to support decision-making and to enhance knowledge discovery. Further analysis of identified patient subgroups by the TURS model may provide clinical insights into discovering patient subtypes.

## Supplementary Material

ocaf171_Supplementary_Data

## Data Availability

The data underlying this article cannot be shared publicly as it contain private information of ICU patients of Leiden University Medical Center, Leiden, The Netherlands.
