# Supplementary material for: Interpretable machine learning for identifying ICU readmission risk in subgroups with probabilistic rules
Source: J Am Med Inform Assoc. 2025 Oct 29;33(3):690–9. doi: 10.1093/jamia/ocaf171 (PMC12981653; doi:10.1093/jamia/ocaf171)
Supplement: ocaf171_Supplementary_Data [file ocaf171_supplementary_data.zip › supp.pdf]

Appendix: Interpretable Machine Learning for Identifying ICU Readmission Risk in Subgroups with Probabilistic Rules

Lincen Yang Ph.D.*[1], Siri L. van der Meijden Ph.D. †[2, 3], Sesmu M. Arbous Ph.D. ‡[2, 4], Matthijs van Leeuwen Ph.D. ‡[1]

[1]Leiden Institute of Advanced Computer Science, Leiden University, Leiden, The Netherlands

[2]Department of Intensive Care Medicine, Leiden University Medical Center, Leiden, The Netherlands

[3]Healthplus.ai B.V., Amsterdam, The Netherlands

[4]Department of Clinical Epidemiology, Leiden University Medical Center, Leiden, The Netherlands

*Corresponding author. Email: l.yang@liacs.leidenuniv.nl. Postal address: Einsteinweg 55 2333 CC Leiden, The Netherlands.

†Co-first author.

‡These authors jointly supervised this work.

Appendix

I: Logistic regression with L_1 regularization

To identify features that are highly associated with the target variable for each individual subgroup, we apply logistic regression with L_1 regularization separately to each patient subgroup. Specifically, the regularization parameter is tuned by five-fold cross-validation with the ROC-AUC (on training data) as the model selection criterion. Note that we normalize the feature matrix for building the logistic regression models (but not for learning the rule set model), as required by the regularization method [1].

Features are grouped by domain experts as being markers for a patient's circulation, coagulation, infection, kidney function, liver function, metabolic function, patient characteristics, or respiratory function. The feature within each group that has the highest association with the target variable is reported.

II: Characterizing deviating features

We next describe how we rank the feature variables based on their deviations. We only report the top-1 deviating feature for each feature group defined above (Section).

Given a feature variable X_j, we denote all values of X_j in our dataset as $\vec{x_j} = \{x_{1j}, ..., x_{nj}\}$. Next, we calculate the "default" mean and standard deviation respectively as

$$u_j = \sum_{i=1}^{n} x_{ij}/n, \quad s_j = \sqrt{\sum_{i=1}^{n}(x_{ij} - u_j)^2/(n-1)}. \tag{1}$$

Further, given a subgroup described by a rule, we denote the indices of patients that meet its condition by the index set I, and we can calculate the *rule mean*

as

$$u'_j = \sum_{i \in I} x_{ij}/|I|, \tag{2}$$

in which $|I|$ denotes the number of elements in the index set I. Consequently, we can now define the *degree of deviation* for a single feature X_j as

$$d(X_j) = abs(u_j - u'_j)/s_j, \tag{3}$$

in which $abs(.)$ denotes the absolute value[1]. Based on the degree of deviation $d(.)$ for each feature, we can rank for a certain subgroup all feature variables and hence discover the most deviating features.

III: Flowchart for reasons of exclusion

We present the flowchart below in Figure 1.

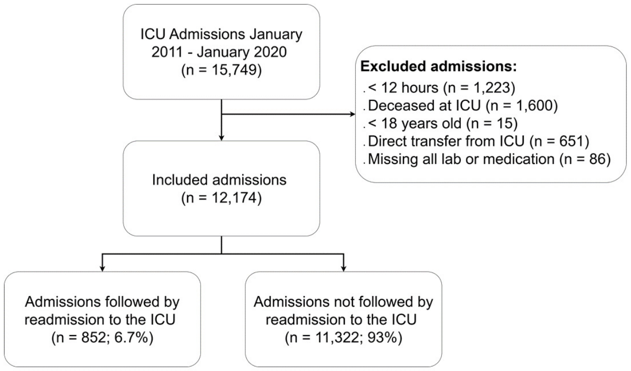

Figure 1: Reasons for exclusion in the data collection.

Abbreviations	Explanation	Location	Row index	Column index	Row index inside
LOS	Length of ICU stay	Table 4	1	3	—
APTT	Activated partial thromboplastin time	Table 4	4	3	—
BE	Base excess	Table 4	7	3	—
CRP	C-reactive protein	Table 4	8	3	—
Specialty: ICCHI	General surgery patient	Table 4	17	3	—
ALAT	Alanine transaminase	Table 4	22	3	—
Priority code 1	Emergency admission	Table 4	25	3	—
ABP	Arterial blood pressure	Table 4	27	3	—
Specialty: ICCTC	Cardiothoracic surgery patient	Table 4	33	3	—
Specialty: ICKNO	Ear-nose-throat surgery patient	Figure 1	1	1	2
Specialty: ICINT	Internal medicine patient	Figure 1	1	1	19
HAS: Via SEH	Patients admitted through emergency room	Figure 1	1	1	20
Specialty: ICCAR	Cardiology patient	Figure 1	1	2	4
Specialty: ICMDL	Gastroenterology patient	Figure 1	1	2	11
Specialty: ICNEU	Neurology patient	Figure 1	2	2	6
Specialty: ICEND	Endocrinology patient	Figure 1	2	2	10
Specialty: ICCHI	General surgery patient	Figure 1	2	2	15
ASAT	Aspartate aminotransferase	Figure 1	3	2	6

Table 1: List of abbreviations and their explanations

IV: Table for abbreviations in the variable names

References

[1] Tibshirani R. Regression shrinkage and selection via the lasso. Journal of the Royal Statistical Society Series B: Statistical Methodology. 1996;58(1):267-88.

[2] Hedges LV, Olkin I. Statistical methods for meta-analysis. Academic press; 2014.

[1] Equation (3) resembles the definition of (the absolute value of) the *Glass' Delta* [2], which is one way of defining the effect size
