# Supplementary material for: Interpretable machine learning for identifying ICU readmission risk in subgroups with probabilistic rules
Source: J Am Med Inform Assoc. 2025 Oct 29;33(3):690–9. doi: 10.1093/jamia/ocaf171 (PMC12981653; doi:10.1093/jamia/ocaf171)
Supplement: ocaf171_Supplementary_Data [file ocaf171_supplementary_data.zip › TF-JAMI250172_Log_Rpt.html]

|  |  |  |  |  |
| --- | --- | --- | --- | --- |
| **File Name:** |  |  | **Status:** Success | **Processed Time:** 23-Sep-25 5:40:26 PM |

---

##### Login Report

| Error Description |
| --- |
| No Error - File moved successfully |
